# Application of a household-based molecular xenomonitoring strategy to evaluate the lymphatic filariasis elimination program in Tamil Nadu, India

**DOI:** 10.1371/journal.pntd.0005519

**Published:** 2017-04-13

**Authors:** Swaminathan Subramanian, Purushothaman Jambulingam, Brian K. Chu, Candasamy Sadanandane, Venkatesan Vasuki, Adinarayanan Srividya, Mohamed S. Mohideen AbdulKader, Kaliannagounder Krishnamoorthy, Harikishan K. Raju, Sandra J. Laney, Steven A. Williams, Ralph H. Henderson

**Affiliations:** 1 Vector Control Research Centre (Indian Council of Medical Research), Indira Nagar, Puducherry, India; 2 Neglected Tropical Diseases Support Center, Task Force for Global Health, Decatur, Georgia, United States of America; 3 Institute of Zoonoses and Vector Control, Department of Public Health, Hosur, Govt. of Tamil Nadu, India; 4 SJL Global Consulting, Seattle, Washington, United States of America; 5 Department of Biological Sciences, Smith College, Northampton, Massachusetts, United States of America; Washington University School of Medicine, UNITED STATES

## Abstract

**Background:**

The monitoring and evaluation of lymphatic filariasis (LF) has largely relied on the detection of antigenemia and antibodies in human populations. Molecular xenomonitoring (MX), the detection of parasite DNA/RNA in mosquitoes, may be an effective complementary method, particularly for detecting signals in low-level prevalence areas where *Culex* is the primary mosquito vector. This paper investigated the application of a household-based sampling method for MX in Tamil Nadu, India.

**Methods:**

MX surveys were conducted in 2010 in two evaluation units (EUs): 1) a hotspot area, defined as sites with community microfilaria prevalence ≥1%, and 2) a larger area that also encompassed the hotspots. Households were systematically selected using a sampling interval proportional to the number of households in the EU. Mosquito pools were collected and analyzed by real-time polymerase chain reaction (qPCR). Two independent samples were taken in each EU to assess reproducibility of results. Follow-up surveys were conducted in 2012.

**Results:**

In 2010, the proportion of positive pools in the hotspot EU was 49.3% compared to 23.4% in the overall EU. In 2012, pool positivity was significantly reduced to 24.3% and 6.5%, respectively (p<0.0001). Pool positivity based on independent samples taken from each EU in 2010 and 2012 were not significantly different except for the hotspot EU in 2012 (p = 0.009). The estimated prevalence of infection in mosquitoes, measured by PoolScreen, declined from 2.2–2.7% in 2010 to 0.6–1.2% in 2012 in the hotspot area and from 0.9–1.1% to 0.2–0.3% in the larger area.

**Conclusions:**

The household-based sampling strategy for MX led to mostly reproducible results and supported the observed LF infection trends found in humans. MX has the potential to be a cost-effective, non-invasive monitoring and evaluation tool with sensitive detection of infection signals in low prevalence settings. Further investigation and application of this sampling strategy for MX are recommended to support its adoption as a standardized method for global LF elimination programs.

## Author summary

Lymphatic filariasis (LF) is one of the world’s foremost debilitating infectious diseases with nearly 800 million people at risk of infection. Given that LF is a mosquito-borne disease, the use of molecular xenomonitoring (MX) to detect parasite DNA/RNA in mosquitoes can serve as a valuable tool for LF monitoring and evaluation, particularly in *Culex* vector areas. We investigated using MX in a low-level prevalence district of Tamil Nadu, India by applying a household-based sampling strategy to determine trap location sites. Two independent mosquito samples were collected in each of a higher human infection hotspot area *(sites with community microfilaria prevalence ≥1%)* and across a larger evaluation area that also encompassed the hotspots. Pooled results showed mostly reproducible outcomes in both settings and a significant higher pool positivity in the hotspot area. A follow-up survey conducted two years later reconfirmed these findings while also showing a reduction in pool positivity and estimated prevalence of infection in mosquitoes in both settings. The utilization of a household-based sampling strategy for MX proved effective and should be further validated in wider epidemiological settings.

## Introduction

Lymphatic filariasis (LF) is a mosquito-borne parasitic disease caused by the filarial worm species *Wuchereria bancrofti*, *Brugia malayi and Brugia timori*. LF is a major public health problem with nearly 800 million individuals at risk of infection in 73 tropical and subtropical countries worldwide [1]. The burden in India alone comprises nearly one-third of the global total. In response, the country’s LF elimination program has scaled up nationally to reach all 255 endemic districts over 20 states and union territories [2,3]. Since 2000, several of these districts have undergone 10–12 annual rounds of mass drug administration (MDA). As of May 2016, 72 districts have successfully passed the first transmission assessment survey (TAS) and qualified for stopping MDA as per World Health Organization (WHO) guidelines [4]. Of the remaining districts, 1 has passed the second TAS, 35 are eligible for conducting the first TAS, and MDA is ongoing in the other 147 districts.

Successful elimination of LF requires close monitoring and evaluation of transmission potential in the endemic area to prevent recrudescence. Various diagnostic tools are available for detecting LF antigen and antibody in the infected population [5,6]. LF infection in mosquito vectors has been largely determined by dissection, staining, and microscopy [7], as well as assays by polymerase chain reaction (PCR) to detect filarial DNA and/or RNA in mosquitoes [8–14]. Molecular xenomonitoring (MX) is the detection of parasite DNA/RNA in mosquitoes and can serve as an alternative method for estimating the infection prevalence in human populations [15,16]. However, implementing MX to evaluate the impact and progress of LF national elimination programs has not yet been adopted as a standard monitoring and evaluation tool, in contrast to its wider success in onchocerciasis control and elimination programs [17].

Progress in the application of MX has been most rapid where *Culex*, common in south and southeast Asia, is the primary vector. Conversely, MX has been constrained in areas where the predominant vectors are *Anopheles*, as in West Africa, or *Aedes*, which prevails in the South Pacific. This is largely due to difficulties in collecting these species. *Culex* is more easily obtained, usually by placing traps in locations thought to be attractive to ovipositing mosquitoes. Although replicable results may result from repeat sampling at the same sites, the arbitrary nature of the selection of sites makes comparisons from different geographic locations problematic. It is preferable for monitoring vector infection that the mosquito collection is done by placing traps in randomly selected sites, but using a systematic sample that can be repeated at different times and at different locations.

The WHO convened meetings in 2002 and 2006 to discuss the application of MX for LF elimination programs [18,19]. In 2009, Pedersen et al. provided a comprehensive review of the field and drew attention to the need for careful considerations of mosquito collection methods in addition to other factors [16]. Also in 2009, an international workshop on MX for LF was hosted by the Vector Control and Research Center (VCRC) in Pondicherry, India. A random sampling method for *Culex* collection was presented that entails selecting a cluster sample of households (HHs) at which gravid traps are placed and a pre-determined number of mosquito pools are collected. This paper summarizes the results of studies by the VCRC utilizing this HH-based sampling strategy in 2010 and 2012 in Thanjavur, a semi-rural district in the state of Tamil Nadu, India.

## Methods

### Ethics statement

This study involved collection of mosquitoes using gravid traps placed outside the households such that it does not interfere with any domestic activities within or around the households. Therefore, there were no ethical issues and all heads of households consented to the placement of the traps.

### Study area

The study was conducted in the Primary Health Center (PHC) of Ammapettai in Thanjavur district, Tamil Nadu, India comprising an area of approximately 40 km^2^ with a population of 19,147 residing in 5,910 households. *Culex quinquefasciatus* is the LF transmitting vector in this PHC. Ammapettai has 18 villages and 15 wards under six health sub-centres and has undergone eight annual rounds of MDA since 1997 –four rounds with diethylcarbamazine (DEC) alone and four rounds with DEC plus albendazole (ALB). MDA was not carried out in 1998, 2005 and 2006 and had been stopped in 2008 after a 2008–2009 mass screening had shown microfilaria (Mf) prevalence was less than 1% and antigenemia (Ag) prevalence was less than 2% in children 2–10 years old, thus meeting the WHO criteria for stopping MDA [2]. Some wards in Ammapettai, however, were identified as residual hotspots where the Mf prevalence was greater than or equal to 1% [2], hereafter called ‘hotspots’ in this study.

### Evaluation units

**Fig 1:**
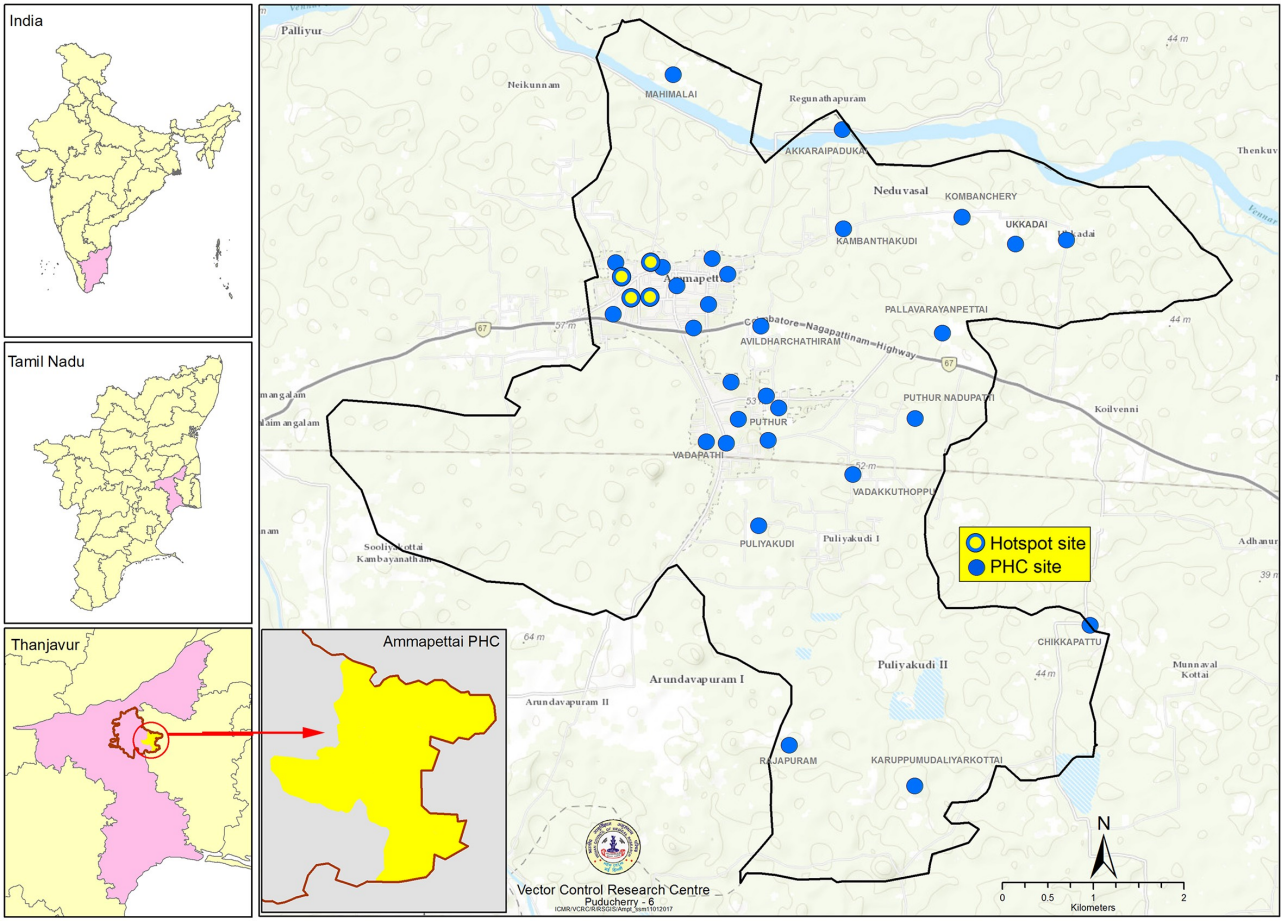
Map of study area, hotspot evaluation unit sites, and PHC evaluation unit sites.

MX surveys were initially carried out between September 2009 and February 2010 in two evaluation units (EUs), which were district subunits and not equivalent to the EUs used for the TAS. The first EU comprised all the hotspot areas where microfilaria prevalence was greater than 1%, as identified in the 2008–2009 mass screening. This hotspot EU consisted of 17 streets under 4 wards in Ammapettai. The second EU consisted of the entire PHC area of Ammapettai, which included the 4 hotspot wards for a total of 33 wards/villages (sites). Fig 1 illustrates the location of Ammapettai within India and distinguishes between hotspot and PHC EU sites in the study.

### Repeat surveys

The 2010 hotspot and PHC surveys were repeated between October 2012 and Jan 2013. All surveys followed the same household selection and mosquito collection procedures outlined further below. Following the 2010 MX surveys, each resident who was found Mf- or Ag-positive in the 2008–2009 mass screening was to be treated with a 12-day course of DEC (6 mg/kg body weight) following the national program guidelines. Of the 369 persons that were positive for Mf or Ag, 303 (82.1%) received treatment between June 10–25, 2010. Replicating the MX study in 2012 was, therefore, intended to demonstrate whether the treatment also reduced the parasite infection load and if the HH-based sampling strategy would detect such change in the EUs.

### Independent samples

Two independent samples were collected for each PHC and hotspot survey to assess the sampling method’s reproducibility of results. Therefore, a total of 4 samples were collected each year (two per EU) and labeled in this study as: 2010 Hotspot (sample 1), 2010 Hotspot (sample 2), 2010 PHC (sample 1), 2010 PHC (sample 2), 2012 Hotspot (sample 1), 2012 Hotspot (sample 2), 2012 PHC (sample 1), and 2012 PHC (sample 2). Independent samples within the same survey (e.g. 2010 Hotspot Survey–samples 1 and 2) were taken no more than 1 month apart in the hotspot area and approximately two months (2010) and one month apart (2012) in the PHC areas. All independent samples were collected during the peak biting season to best control the impact of environmental variables. Each sample took a median of two nights in the hotspot EU and three nights in the PHC EU to complete a collection of 2 pools.

### Sample size

For each independent sample, the aim was to collect pools of 25 mosquitoes from 200 HH trap locations for a total sample size of 5,000 mosquitoes. These parameters were based on a target infection prevalence rate of 0.5%, which has been previously recommended for *Culex* mosquitoes [20]. Other sources have suggested a target rate of 0.25% [12,19]. Our study was, therefore, powered to correctly detect at least 75% of the time if the true prevalence is less than 0.25%, while failing only 5% of the time to detect if the true prevalence is greater than 0.5% (i.e. alpha error). Given the low target prevalence rate, pool sizes of 25 mosquitoes were estimated to have negligible measurement bias and deemed appropriate for this study [21].

### Household selection

HHs were randomly selected in each EU as trap location sites. Random selection was done by first calculating a fixed sampling interval proportional to the total number of HHs in the EU to meet the 200 HH target. After enumerating each HH in each village/ward, a random HH was chosen as the first HH in the first village/ward. Every subsequent HH was then selected by adding the fixed sampling interval to the enumerated HHs. Separate sampling intervals were calculated for the PHC and hotspot EUs. The sampling intervals were not proportional to the size of each individual village/ward nor were they reset at the start of each village/ward. On average, 7 HHs were selected per village/ward in the PHC EU and 12 HHs per street in the 4 hotspot EU. As a result, a total of 231 HHs (33 villages/wards x 7 HHs) were selected in the PHC EU and 204 households (17 streets x 12 HHs) in the hotspot EU for placing the mosquito traps.

This HH sampling strategy may also employ a two-staged cluster design, where the first stage involves systematically selecting only certain clusters from the EU [22]. Due to the smaller geographic area in this study, all the clusters (i.e. villages/wards) from the hotspot and PHC EUs were included for HH selection.

### Mosquito collection

A modified version of the CDC Gravid trap (Model 1712, John W. Hock Co. USA) was placed each evening close to the selected HHs [23,24]. Mosquito traps and nets containing the catch were collected each morning and returned to a central laboratory where the mosquitoes were killed by freezing before being sorted for *Culex quinquefasciatus* that were either gravid, semi-gravid, or showed evidence of having recently ingested a blood meal. Mosquitoes that met these criteria were then stored together in pools of 25 mosquitoes (fewer if the trap yield was insufficient with a minimum of 5 mosquitoes per pool) after drying them at 95°C for a minimum of 15 minutes for later qPCR analysis. A single trap at the selected HH was used to collect all pools required at that site. If on any given night the trap yield was insufficient to complete a full pool of 25, mosquitoes from the subsequent night’s yield were added to complete the pool. If more than a full pool was collected, extra mosquitoes were allocated to the next pool. All excess mosquitoes beyond completion of the required number of pools were discarded. Traps were set each day at the same HH locations until the required number of pools had been obtained, or for a maximum of three nights. The oviposition bait was also replaced daily with a fresh batch prior to fixing the trap.

An adult HH resident was asked for permission to set the traps outside the HH and field teams placed them in areas less prone to thievery or obstruction. No denials were experienced despite the unpleasant odors of the bait, presumably because residents welcomed the fact that the traps removed mosquitoes from the surrounding area. Batteries, which ran the trap fans, were recharged each day and no instances of trap disruption were encountered (e.g. vandals stealing the batteries).

### DNA extraction and real-time PCR analysis

DNA extraction of mosquito pools and real-time PCR analysis for detecting *W*. *bancrofti* DNA in individual pools was performed at the VCRC using the BB-grinding method to macerate the mosquito pools [13], the optimized Qiagen DNA extraction method [25], and the qPCR assay [11] described in previously published reports.

### Data analysis

Comparisons of pool positivity (number of pools positive for filarial parasite DNA over total number of pools screened) and 95% confidence intervals were conducted with chi-square tests for equality of proportions (without Yates continuity correction) using R (version 3.3.2). The maximum likelihood estimate and its 95% confidence intervals of *W*. *bancrofti* infection prevalence in mosquitoes were made using the PoolScreen software (version 2.0.3) [21,26]. GPS coordinates for trap locations were collected using the Dell Axim X51 personal digital assistant and mapped using ArcGIS (version 10.2.1) (ESRI, Redlands, CA).

## Results

In the hotspot EU (Table 1), an average of 5,012 total mosquitoes per sample (range: 4,867–5,175) was collected from 207 HHs (trap locations). The mean number of *Culex* (gravid, semi-gravid, and bloodfed) per pool varied between 24.5–25.0 for the samples in 2010, and 23.7–24.0 in 2012. More than 90% of the pools had 21–25 mosquitoes per pool. The qPCR result for one pool in three of the four independent samples was indeterminate and excluded in the analysis.

**Table 1:** Pool positivity and estimated prevalence of filarial DNA in the hotspot EU, 2010 and 2012.

Year	Survey (sample)	Household trap sites	Pools collected	Mosquitoes collected	Pool size mean and SD (range)	Positive pools	% Positive pools [95% CI]	*W*.*bancrofti* DNA detection prevalence in mosquitoes^1^ [95% CI]
**2010**	**Hotspot (sample 1)**	207	207	5175	25.0 ± 0.0 (25–25)	102	49.3% [42.5, 56.0]	2.7% [2.1, 3.3]
	**Hotspot (sample 2)**	207	206	5045	24.5 ± 2.4 (9–25)	88	42.7% [36.2, 49.5]	2.2% [1.7, 2.8]
**2012**	**Hotspot (sample 1)**	207	206	4867	23.7 ± 3.4 (8–25)	50	24.3% [18.9, 30.1]	1.2% [0.8, 1.6]
	**Hotspot (sample 2)**	207	206	4962	24.0 ± 2.5 (10–25)	29	14.1% [10.0, 19.5]	0.6% [0.4, 0.9]

^**1**^ Maximum likelihood estimate using PoolScreen.

**Fig 2:**
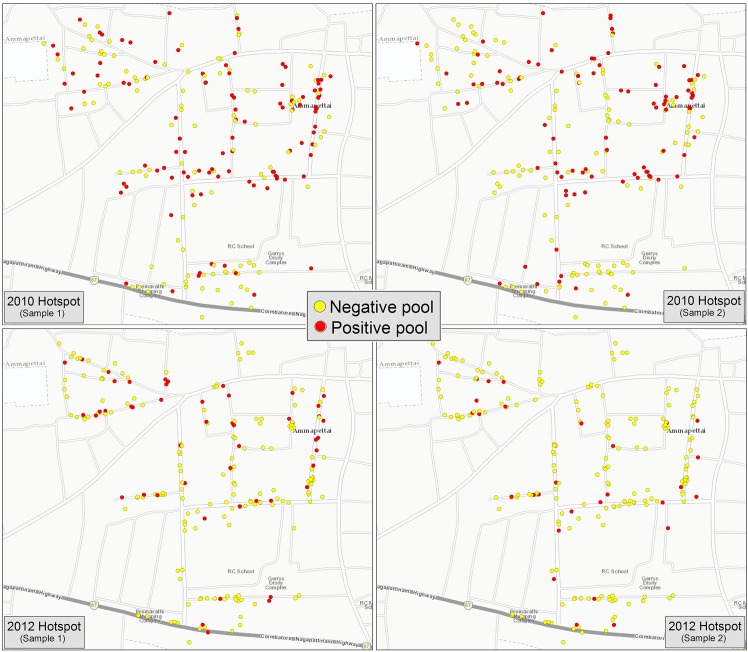
Map of positive and negative pools by household location in the Hotspot evaluation unit, 2010 and 2012.

Fig 2 maps the HH locations of all positive and negative pools for each hotspot EU sample in 2010 and 2012. In 2010, the proportion of positive pools was 49.3% (102/207) in the first sample and 42.7% (88/206) in the second sample. The PoolScreen estimated prevalence of infection was 2.7% and 2.2%, respectively. In 2012, the proportion of positive pools was 24.3% (50/206) in the first sample and 14.1% (29/206) in the second sample. The PoolScreen estimated prevalence of infection was 1.2% and 0.6%, respectively.

For the PHC EU (Table 2), the average number of mosquitoes collected per sample was 5,311 (range: 5,094–5,437) from 231 trap locations. The mean pool size varied between 23.2 and 23.5 for the samples in 2010, and 22.0–23.3 in 2012. Between 81–84% of the pools had 21–25 mosquitoes per pool. Although 231 pools were collected in the second 2010 sample, the qPCR result for one mosquito pool was indeterminate and, therefore, excluded in the analysis. Results for all pools in the other samples were valid and analyzed.

**Table 2:** Pool positivity and estimated prevalence of filarial DNA in the PHC EU, 2010 and 2012.

Year	Survey (sample)	Household trap sites	Pools collected	Mosquitoes collected	Pool size mean and SD (range)	Positive pools	% Positive pools [95% CI]	*W*.*bancrofti* DNA detection prevalence in mosquitoes ^1^ [95% CI]
**2010**	**PHC (sample 1)**	231	231	5437	23.5 ± 3.2 (10–25)	54	23.4% [18.4, 29.2]	1.1% [0.8, 1.5]
	**PHC (sample 2)**	231	230	5329	23.2 ± 3.5 (9–25)	41	17.8% [13.4, 23.3]	0.9% [0.6, 1.2]
**2012**	**PHC (sample 1)**	231	231	5094	23.3 ± 3.7 (8–25)	15	6.5% [4.0, 10.4]	0.3% [0.2, 0.5]
	**PHC (sample 2)**	231	231	5385	23.0 ± 5.7 (2–25)	12	5.2% [3.0, 8.9]	0.2% [0.1, 0.4]

^1^ Maximum likelihood estimate using PoolScreen.

**Fig 3:**
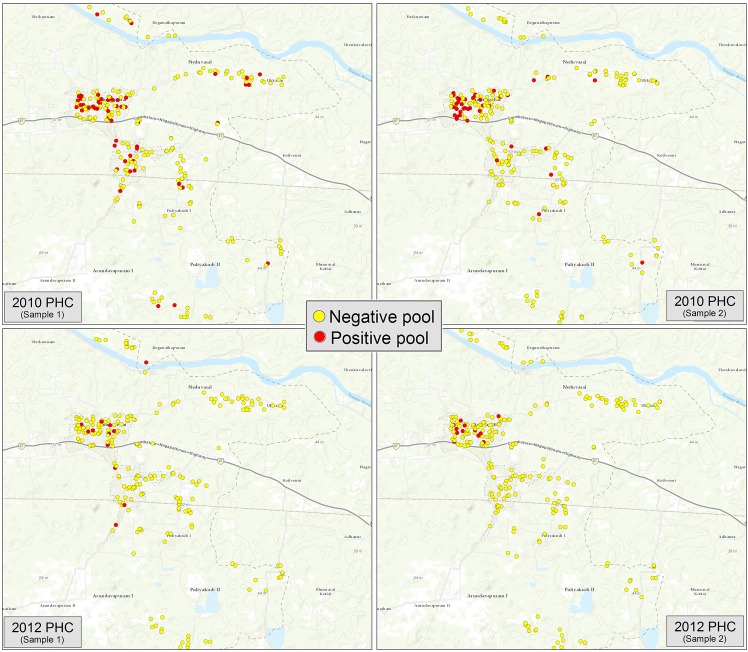
Map of positive and negative pools by household location in the PHC evaluation unit, 2010 and 2012.

Fig 3 maps the HH locations of all positive and negative pools for each PHC EU sample in 2010 and 2012. Pool positivity in 2010 was 23.4% (54/231) in the first sample and 17.8% (41/230) in the second sample. PoolScreen results were 1.1% and 0.9%, respectively. In 2012, pool positivity was 6.5% (15/231) in the first sample and 5.2% (12/231) in the second sample. PoolScreen results were 0.3% and 0.2%, respectively.

Table 3 compares each pair of independent samples taken from the same EU. In testing the equality of proportions, no statistically significant differences (p>0.05) in pool positivity were detected between the 2010 hotspot samples or either of the 2010 and 2012 PHC samples. However, a significant difference was observed between the samples in the 2012 hotspot EU (p = 0.009). The results also show that the pool positivity of the second samples were lower than that in the first samples for each survey. The upper 95% confidence interval limits suggest this variability is greater in the hotspot samples than the PHC ones.

**Table 3:** Comparison of pool positivity between independent samples for hotspot and PHC surveys, 2010 and 2012.

Year	Survey (sample)	Positive pools (%)	p-value	95% CI for difference of proportions
**2010**	**Hotspot (sample 1)**	102/207 (49.3%)	0.181	[-0.03, 0.16]
	**Hotspot (sample 2)**	88/206 (42.7%)		
	**PHC (sample 1)**	54/231 (23.4%)	0.141	[-0.02, 0.13]
	**PHC (sample 2)**	41/230 (17.8%)		
**2012**	**Hotspot (sample 1)**	50/206 (24.3%)	0.009	[0.03, 0.18]
	**Hotspot (sample 2)**	29/206 (14.1%)		
	**PHC (sample 1)**	15/231 (6.5%)	0.552	[-0.03, 0.06]
	**PHC (sample 2)**	12/231 (5.2%)		

Pool positivity in 2012 was significantly lower than in 2010 for all hotspot and PHC samples (Table 4). The PoolScreen estimated prevalence of infection in mosquitoes was also reduced by more than half in each sample for both surveys over the two years. The exact significance of this decline, however, could not be calculated given the current configuration of the PoolScreen program.

**Table 4:** Comparison of pool positivity and estimated prevalence of filarial DNA between 2010 and 2012 in hotspot and PHC surveys.

Survey (sample)	2010 Positive pools (%)	2012 Positive pools (%)	p-value	2010 *W*.*bancrofti* DNA detection prevalence in mosquitoes ^1^ [95% CI]	2012 *W*.*bancrofti* DNA detection prevalence in mosquitoes ^1^ [95% CI]
**Hotspot (sample 1)**	102/207 (49.3%)	50/206 (24.3%)	<0.0001	2.7% [2.1, 3.3]	1.2% [0.8, 1.6]
**Hotspot (sample 2)**	88/206 (42.7%)	29/206 (14.1%)	<0.0001	2.2% [1.7, 2.8]	0.6% [0.4, 0.9]
**PHC (sample 1)**	54/230 (23.5%)	15/231 (6.5%)	<0.0001	1.1% [0.8, 1.5]	0.3% [0.2, 0.5]
**PHC (sample 2)**	41/230 (17.8%)	12/231 (5.2%)	<0.0001	0.9% [0.6, 1.2]	0.2% [0.1, 0.4]

^**1**^ Maximum likelihood estimate using PoolScreen.

## Discussion

MX surveys, using a systematic sampling of HHs for placing gravid traps, provided an efficient method to collect approximately 5,000 *Culex* mosquitoes in pools of 25 mosquitoes from over 200 HHs. This HH-based sampling strategy was successfully implemented in two EUs and independent samples within each survey largely showed reproducible (i.e. no statistically significant difference) results in terms of pool positivity. The one exception was the 2012 hotspot survey and in general, the hotspot surveys had more sample variability than the PHC surveys. The exact reason behind this trend remains uncertain as the samples were independent and randomly selected. It is also unclear why the second sample of each survey pair had lower pool positivity but perhaps the one- to two-month time gap between sample collections was a contributing and limiting factor. Our study also provided early evidence that a HH-based sampling method can obtain consistent estimates of the prevalence of filarial DNA measured by the PoolScreen technique. From these initial results, it appears that determining the parasite load in the vector population has great potential for the monitoring and evaluation of LF elimination programs where *Culex* is the primary vector.

This HH-based sampling method for MX also produced results consistent with the previously observed LF infection trends found in humans in Ammapettai. In all the 2010 and 2012 MX samples, the hotspot EU had higher pool positivity and estimated prevalence of filarial infection than the overall PHC area. This finding corroborates the human infection rates determined in the 2008–2009 mass screening survey where Mf prevalence was 1.4% (0.9–1.96%) in the hotspot areas and 0.4% (0.30–0.52%) in the overall PHC area. The human infection rate in the screening survey was significantly higher in the hotspots than in the overall PHC (P<0.001). Since Mf- or Ag-positive individuals were also treated with DEC following the 2010 MX surveys, the decline in pool positivity rates and filarial DNA in the 2012 MX surveys most likely reflects the impact of this treatment. The MX results, therefore, supplied an indirect indicator of LF infection in humans, which can be invaluable for transmission assessment and implementing follow-up interventions.

HH-based MX surveys would be particularly helpful in conjunction with the TAS, the method currently recommended by WHO for stopping MDA and post-MDA surveillance [4]. The WHO recommends that an area passes the TAS when filarial antigen prevalence among first and second grade children is less than 1% by ICT (with a 95% CI of less than 2%) [4], a threshold below which transmission is thought to be no longer sustainable in *W*. *bancrofti* areas. In Sri Lanka, however, Rao et al. have shown that the TAS did not identify areas shown by MX to have persisting low levels of transmission as evidenced by the continuing prevalence of filarial DNA in mosquitoes over time [27]. Other studies concluded that MX surveys were more sensitive than Mf testing in humans [12,22]. As such, MX surveys can be a strong complement to the TAS for both stopping MDA and post-MDA surveillance, particularly where low levels of infection persist and are less detectable through human-based surveys by ICT or Mf testing. This application is relevant to Ammapettai and other areas in India where similar MX studies have begun in districts which either passed the TAS once, passed the TAS twice, or passed the first TAS but the number of Ag-positives was very close to the critical cut-off threshold.

MX surveys may also become a more attractive option for post-MDA surveillance as programmatic resources for LF erode, if not disappear, after MDA is discontinued. There may be minimal capacity and little incentive to continually repeat TAS multiple years after drug distribution has stopped. Conversely, there will be plenty of work remaining for entomology staff in assessing threats from other mosquito-borne diseases. Integrating LF to such pre-established monitoring responsibilities may be a more feasible surveillance approach in the long-run than trying to repeat an LF-specific survey such as TAS. With many countries transitioning into post-MDA surveillance mode, it is critical that MX sampling strategies and baseline measures are quickly established. This includes the validation and possible revision of MX thresholds for LF transmission measured through filarial DNA [19].

Selecting adequate EU boundaries is a critical decision for MX surveys and subject to the general limitations of other cluster sample surveys. Larger EUs provide significant cost and resource efficiencies, particularly in a country like India where many EUs need to be evaluated to cover the entire LF transmission area. However, this increases the risk of missing pockets of transmission given that infected areas may be highly focal following MDA. The MX study here used a relatively small EU but was successful in detecting ongoing hotspots of LF transmission. Larger EUs in other MX studies have also succeeded with general results and findings similar to the ones discovered here [22]. It is, therefore, recommended that the epidemiological characteristics and infection risks within the EU are consistent and boundaries are not solely determined by population or geographic size. Further research, however, is required to understand the EU limits for which MX using this HH-based sampling strategy is appropriate, as well as an examination of the logistics, feasibility and cost implications.

Implementation costs are another crucial element to consider for LF monitoring and evaluation tools. Given the proper training and resources, MX surveys may be arguably more cost-effective and less onerous to execute than the TAS or other population-based surveys. The cost advantages of MX also include indirect costs such as the efforts needed for permissions and consent. In this study, permissions to place the traps were relatively easy. Permissions for human studies can be more difficult where the Ministry of Education, school principals and parents may all need to consent and wider community sensitization is required. Collecting mosquitoes is also less intrusive than collecting blood from children and does not generate resistance when repeated sampling is done. In fact, collecting and essentially removing mosquitoes was often perceived positively by households where the traps were placed.

The current study used a total mosquito samples of approximately 5,000, collecting one pool of 25 mosquitoes from each of 200 HHs. The MX work in Sri Lanka used samples of approximately 7,500 mosquitoes, collecting two pools of 25 mosquitoes from each of 150 HHs or 4 pools from 75 HHs [22]. Larger samples may be required for assessing really low prevalence of infection in mosquitoes (on the order of 0.3% or below) at which *Culex* transmission in many environments appears to be difficult. Collecting even larger numbers of mosquitoes would further improve precision, but *Culex* is not always abundant and increasing the number of pools per site will be challenging in some areas. On the other hand, the Sri Lanka work also confirmed that sampling from 75 or 150 HHs is not statistically inferior to sampling from 300 HHs. Reducing the number of mosquito collection sites would vastly improve costs, feasibility, and the overall efficiency of a HH-based MX sampling strategy.MX is admittedly not easy to introduce into programs without PCR or entomological expertise. Given the present capacity in many areas, MX may be better used to assess special situations in which doubt remains about LF infection levels. Nevertheless, simplifications of PCR analysis are being rapidly introduced and the entomological skills required to identify gravid or semi-gravid mosquitoes and to place traps at suitable locations near the selected HHs can be trained or externally provided. Training programs to build MX capacity and resources would also certainly expand should MX progress into a more standardized LF monitoring and evaluation tool. Utilizing regional reference centers with MX resources and expertise offers another option if developing local capacity proves unfeasible.

Extending a HH-based MX sampling approach for other vector species poses difficulties primarily due to challenges in trapping. *Anopheles* mosquitoes require invasive indoor HH trapping procedures and even these procedures have quite low yields. They are not as efficient as other vectors, however, and a transmission threshold of 1% has been suggested as opposed to 0.25% or 0.50% for *Culex* [19,20], although any threshold is highly dependent on corresponding biting rates and annual transmission potentials. Regardless, this difference potentially provides the opportunity to reduce the number of *Anopheles* mosquitoes needed to around 2,500 depending on the magnitude of the prevalence to be detected. Despite the limitations of MX with *Anopheles*, a recent study in northern Nigeria dissected mosquitoes from knock-down collections and clearly showed that the distribution of long lasting insecticide-treated bednets reduced overall *W*.*bancrofti* DNA detection prevalence in mosquitoes from 0.32%, measured when MDA alone was used, to zero when bednets were added [28]. Although these results were obtained through dissection, which admittedly is not as sensitive as qPCR, they still provide convincing evidence for the significant reduction of infection prevalence in an *Anopheles* area.

*Aedes* mosquitoes present a further challenge. Since they are highly efficient vectors, LF transmission can be sustained at quite low prevalence. Therefore, a threshold of less than 0.1% has been suggested for assessing infection in *Aedes* mosquitoes [19]. This, however, implies the need for very large mosquito samples. An MX study conducted in American Samoa, collected over 22,000 female mosquitoes using BG Sentinel traps (Biogents AG, Regensburg, Germany), most of which were the area’s primary LF vector, *Ae*. *Polynesiensis* [29]. While Schmaedick et al. concede that MX for programmatic purposes in *Aedes* areas will require more efficient collection methods and further research, the results suggest that monitoring LF in *Aedes* could still prove useful as a supplement or alternative to monitoring in humans to identify areas where infections may exist. A follow-up study in American Samoa confirmed a statistically significant association between MX and human seroprevalence data, which further demonstrates the potential of MX as a long term surveillance strategy to locate transmission hotspots [30].

Our study included PoolScreen results to estimate LF infection prevalence in mosquitoes. PoolScreen has only recently been used to analyze mosquito samples for the prevalence of LF, which is a more focal disease than onchocerciasis for which PoolScreen has been more frequently applied. Given the low prevalence levels being assessed in this study and presumably other post-MDA settings, PoolScreen appears quite practical and reasonable for LF surveillance efforts according to its statistical parameters [21]. Extending PoolScreen’s capability to definitively compare changes in infection rates across independent random samples and calculate design effects for cluster surveys will further validate its program effectiveness and help mitigate remaining statistical concerns.

## Conclusions

A method for sampling *Culex* mosquitoes for the analysis of *W*. *bancrofti* infection using gravid traps placed at systematically selected HH sites was successfully implemented across multiple surveys in two separate EUs. The results mostly showed no significant difference in repeat samples and were consistent with the estimated trends for human LF infection in the same area. The overall sampling strategy and results were also in agreement with a larger HH-based MX study in Sri Lanka [22].

As mosquito trapping and qPCR methodologies improve, so will the prospect of utilizing MX for other vector species. Additional work is needed to compare this MX approach to other survey methodologies for assessing LF prevalence in communities, particularly the TAS which is currently recommended by WHO for stopping MDA and post-MDA surveillance decision-making. Statistically, further research will help extend the approach in terms of assessing minimal sample sizes, clustering effects, and epidemiological constraints in different ecological areas. Finally, from an operational standpoint, it will be useful to examine modifications to the HH sampling method that might improve cost-effectiveness and reduce labor requirements. Addressing all these factors while continuing the application of MX in programmatic settings will undoubtedly speed up its adoption into a more standardized and robust LF evaluation tool.
